# Patient Expectations and Satisfaction in Pediatric Orthopedics

**DOI:** 10.1007/s12178-023-09869-5

**Published:** 2023-09-20

**Authors:** Alejandro Cazzulino, Katherine Bach, Rafael Cordero, Ishaan Swarup

**Affiliations:** 1 Department of Orthopedic Surgery, University of California San Francisco, 500 Parnassus Ave. Millberry Union MU 320 W, San Francisco, CA USA; 2 Ponce Health Sciences University, Ponce, Puerto Rico; 3 Department of Orthopedic Surgery, University of California San Francisco Benioff Children’s Hospital Oakland, Oakland, CA USA

**Keywords:** Pediatrics, Expectations, Satisfaction, Tools, Surveys

## Abstract

**Purpose of Review:**

The purpose of the current review is to analyze the current literature regarding the tools available to evaluate patient expectations and satisfaction. There have been an increasing number of tools that have been developed and validated for various orthopedic procedures. Despite the growing number of tools, there are a limited number of tools available for pediatric patients.

**Recent Findings:**

Several tools have been developed in orthopedics to evaluate patient expectations. However, there are no tools that have been validated in the pediatric population. In addition, pediatric patient expectations should be collected in conjunction with parent/caregiver expectations. Although not specifically validated for pediatric patients, there are several tools available that may pertain to pediatric patients including the HSS ACL Expectations Survey, HSS Shoulder Expectations Survey, HSS Knee Surgery Expectations Survey, HSS Foot and Ankle Surgery Expectation Survey, Sunnybrook Surgery Expectations Survey, Musculoskeletal Outcomes Data Evaluation and Management System (MODEMS) Instruments, Quick DASH, and DASH.

In terms of patient satisfaction, there are even fewer tools available. Several tools have been developed to evaluate patient satisfaction and five additional tools within orthopedics. Of these tools, there are two that have been validated for pediatric patients: The Swedish Parents Satisfaction Questionnaire and the Scoliosis Research Society-22.

**Summary:**

There are a growing number of tools to evaluate patient’s expectations and satisfaction in the orthopedic literature. Given most of these tools pertain to adult patients, there is a need for further development of tools specifically validated for pediatric patients and their parents/caregivers. Through the measurement of expectations and satisfaction, medical professionals can hope to improve satisfaction and outcomes.

## Introduction

Beginning in the early 1990s, studies have attempted to quantify patient expectations before undergoing elective orthopedic procedures through the generation of surveys. The most robust body of literature is centered around total joint replacements [1••, 2–5]. The power of these studies relies upon patient generated expectations that are included in the surveys, which are independent of physician generated expectations. These studies have been expanded to other fields of orthopedics including shoulder surgery [6], hip preservation [7], foot and ankle [8], ACL reconstruction [9], distal radius [10] and cervical [11] and lumbar [12] spine surgery [13, 14]. These patient centered surveys provide insight into what patients expect as an outcome of their surgery, which is critical in a field dominated by elective surgery. Additionally, patient expectations prior to undergoing surgery have been linked to outcomes [15–19], which will be discussed in detail later in this article. Although there is a robust body of literature evaluating patient expectations in the adult population, there is limited evaluation of patient and caregiver expectations in the pediatric population.

As healthcare moves from volume-based to quality-based care, the importance of patient’s expectations prior to undergoing elective orthopedic procedures has increased. Hospital and physician quality metrics are now linked to patient satisfaction [16, 20–22]; therefore, any associated variables warrant further investigation. It is imperative that physicians preoperatively discuss patient’s expectations to ensure that the patient and physician expectations are aligned, given aligned expectations can help to improve outcomes and satisfaction [3, 15, 18, 19, 23]. As with expectations in general, there is a similar lack of literature regarding the relationship between expectations and satisfaction in the pediatric population.

A unique situation in the pediatric patient revolves around the multiple sets of expectations, those of the patient and those of the caregivers, which may differ from each other. This poses a unique challenge for providers given caregivers have been shown to have higher expectations than the patient in pediatric surgeries [24•]. Therefore, understanding both the patient and the caregiver expectations prior to surgery is essential to achieve patient satisfaction and ensure successful outcomes. This study aims to provide an updated summary of tools for evaluating patient expectations and satisfaction, as well as provide a review of the current literature regarding the relationship between expectation, outcomes, and satisfaction in the pediatric orthopedic population.

## Measuring Expectations and Satisfaction in Pediatric Orthopedics

There currently exist a myriad of tools available to measure patient expectations in orthopedic surgery, ranging from study-specific custom questionnaires to validated expectation instruments with reliability data. The majority of patient expectation surveys are surgical procedure or anatomic location specific; however, there are two broadly applicable and validated surveys that can be used for any type of orthopedic procedure. A subset of the patient expectation literature also relies on currently existing clinical outcome measures that are modified to assess patient expectations prior to surgery. All of the validated tools are administered as patient-completed questionnaires. Additionally, multiple studies peformed qualitative patient interviews with open-ended responses to create patient-generated expectation surveys. Topics queried include expectations regarding pain, physical function, recovery time, cosmesis, as well as social and psychosocial factors. A summary of the available tools is summarized in Table 1.

**Table 1:** Summary of patient expectation tools

Patient expectation assessment in orthopedic surgery					
Validated surveys and modified clinical outcome scores					
Assessment tool	Subspecialty	Population	Administration method	Item scoring method	Pediatric validation
HSS Hip Replacement Expectations Survey	Arthroplasty	Adults	Self-administered questionnaire	Adjectival items (5 categories)	No
HSS Knee Replacement Expectations Survey	Arthroplasty	Adults	Self-administered questionnaire	Adjectival items (5 categories)	No
HSS Knee Surgery Expectations Survey	Sports	Adults/Peds	Self-administered questionnaire	Adjectival items (5 categories)	No
New Knee Society Score - Expectations Domain	Arthroplasty	Adults	Self-administered questionnaire	Adjectival items (5 categories)	No
HSS ACL Expectations Survey	Sports	Adults/Peds	Self-administered questionnaire	Adjectival items (5 categories)	No
HSS Shoulder Expectations Survey	Sports	Adults/Peds	Self-administered questionnaire	Adjectival items (5 categories)	No
HSS Cervical and Lumbar Spine Surgery Expectations Surveys	Spine	Adults	Self-administered questionnaire	Adjectival items (5 categories)	No
HSS Foot and Ankle Surgery Expectations Survey	F&A	Adults/Peds	Self-administered questionnaire	Adjectival items (5 categories)	No
Sunnybrook Surgery Expectations Survey	Broadly applicable	Adults/Peds	Self-administered questionnaire	Adjectival items (4 or 5 categories)	No
Musculoskeletal Outcomes Data Evaluation and Management System (MODEMS) Instruments	Broadly applicable	Adults/Peds	Self-administered questionnaire	Adjectival items (5 categories)	No
Western Ontario and McMaster Universities Arthritis Index (WOMAC)	Arthroplasty	Adults	Self-administered questionnaire	Adjectival items (4 categories)	No
Oxford Knee Score	Arthroplasty	Adults	Self-administered questionnaire	Adjectival items (5 categories)	No
Oxford Hip Score	Arthroplasty	Adults	Self-administered questionnaire	Adjectival items (5 categories)	No
QuickDASH	Upper extremity	Adults/Peds	Self-administered questionnaire	Adjectival items (5 categories)	No
DASH	Upper extremity	Adults/Peds	Self-administered questionnaire	Adjectival items (5 categories)	No
NAAS Lumbar Spine Questionnaire	Spine	Adults	Self-administered questionnaire	Skewed Likert items (5 categories)	No
KOOS	Arthroplasty	Adults	Self-administered questionnaire	Adjectival and Likert items (5 to 7 categories)	No
International Knee Documentation Committee Subjective Knee Form	Arthroplasty	Adults	Self-administered questionnaire	Adjectival items (5 categories)	No
Historical Leisure Activity Questionnaire	Broadly applicable	Adults	Self-administered questionnaire	Open-ended responses concerning frequency of activity participation	No
Functional Questionnaire of Hannover for Osteoarthritis	Arthroplasty	Adults	Self-administered questionnaire	3 response questions	No
Knee Society Pain Score	Arthroplasty	Adults	Self-administered questionnaire	7 response questions	No
Total Hip Arthroplasty Outcome Evaluation Questionnaire	Arthroplasty	Adults	Self-administered questionnaire	Combined open-ended, adjectival, limited response option, and VAS	No
Schedule of the Individual Quality of Life-Direct Weights	Broadly applicable	Adults	Self-administered questionnaire	10 centimeter VAS	No

For arthroplasty patients, validated expectation surveys include the Hospital for Special Surgery (HSS) Hip Replacement Expectation Survey [4], the HSS Knee Replacement Expectation Survey [5], and the New Knee Society Scoring System—Expectations Domain [25]. Clinical outcome measures that have been modified to assess patient expectations surrounding total joint replacements include the Oxford Knee Score [26], the Oxford Hip Score [26], the KOOS [27], the International Knee Documentation Committee Subjective Knee Form [28], the Functional Questionnaire of Hannover for Osteoarthritis [29], the Knee Society Pain Score [30], and the Total Hip Arthroplasty Outcome Evaluation Questionnaire [31]. The subspecialties of the shoulder, foot and ankle, and spine, each have validated expectation surveys, namely the HSS Shoulder Expectations Survey [6], the HSS Foot and Ankle Survey [8], and the HSS Cervical and Lumbar Spine Expectation Surveys [11, 12], respectively. Expectations for spine surgery can also be evaluated with a modified NASS Lumbar Spine Questionnaire [32]. Surveys that are broadly applicable to patients undergoing any type of surgery include the Sunnybrook Surgery Expectations Survey [33] and the Expectations Domain of the Musculoskeletal Outcomes Data Evaluation and Management System (MODEMS) Instruments [34]. Similarly, broad clinical outcomes measures such as the WOMAC [35], QuickDASH [36], Disability of the Arm, Shoulder, and Hand (DASH) [37], and Historical Leisure Activity Questionnaire [38] can be adapted to assess patient expectations.

Even with all of these potential tools for the assessment of patient expectations, there are currently no tools to specifically evaluate parent and child expectations in pediatric orthopedic surgery. There are a limited number of clinical tools for assessing patient expectations that would be applicable to children and adolescents undergoing surgeries, such as the HSS Knee Surgery Expectation Survey [5], the HSS ACL-Expectations Score [9], the HSS Shoulder Expectations Survey [6], and the DASH/QuickDASH, but a review of the literature reveals a paucity of tools geared toward the pediatric population given these studies focus on patients greater than 18 years old. This is not altogether surprising, as children would likely have difficulty with the abstract thinking necessary for detailing expectations regarding a surgical intervention. However, there is ample opportunity for the development of expectation surveys for parents with children undergoing orthopedic surgical procedures. Research has shown a strong level of agreement between child and parent responses on outcome instruments after surgery [24•], which provides some credibility for the use of parent proxies in pediatric expectation and satisfaction literature. There also exists a need to develop additional measures for non-English speaking patients, as there are few validated measures for this patient population [39].

Although there is widespread agreement that satisfaction is an important outcome measure following orthopedic surgery, there are very few validated and reliable tools available for clinicians, and the majority of studies do not use a standardized assessment of patient satisfaction. Adding to the challenge is the complexity of satisfaction itself, and the importance of distinguishing between satisfaction with the outcome of care and satisfaction with the process of care, including factors often beyond the surgeon’s control such as cost, inconvenience, wait and visit times, and hospital environment. Assessment tools that are widely used in healthcare systems include the National Research Corporation (NRC) Picker patient satisfaction tool, the Consumer Assessment of Healthcare Providers and Systems instrument, and the Press Ganey Survey [40].Beyond these general tools, the vast majority of current research studies evaluating satisfaction employ either the Visual Analogue Scale-Satisfaction (VAS), a 5- or 7-point Likert scale, or study-specific custom questionnaires. For example, a systematic review of literature regarding patient satisfaction after orthopedic interventions of the hand found that 14 out of 17 articles relied on at least one of these three types of assessment [41]. Similarly, researchers interested in determinants of patient satisfaction after ACL surgery used a 1–10 ordinal scale for two separate questions, “how satisfied are you with your outcome” and “how satisfied are you with the process of treatment,” in an attempt to distinguish between the aforementioned different elements of satisfaction [42]. Although study-specific custom satisfaction questionnaires and yes/no satisfaction surveys are unvalidated [43], the use of the VAS-Satisfaction has been shown to have good validity and reliability in the total hip arthroplasty population [44]. The available tools are summarized in Table 2.

**Table 2:** Summary of patient satisfaction tools

Patient satisfaction measurement tools			
	Procedures	Population	Pediatric validation
General satisfaction measurement tools			
National Research Corporation (NRC) Picker Patient Satisfaction Tool	Variable	Adults	No
Consumer Assessment of Healthcare Providers and Systems (CAHPS)	Variable	Adults	No
Press Ganey Survey	Variable	Adults	No
Visual Analogue Scale - Satisfaction (VAS)	10 cm VAS between “no satisfaction” and “extreme satisfaction”	Adults	No
Five- or Seven-Point Likert Scale	Ordinal scale	Adults	No
Study-Specific Custom Questionnaires	Variable	Adults	No
Swedish Parent Satisfaction Questionnaire	63 question survey	Parents/caregivers	Yes
Satisfaction measurement tools in surgery/orthopedic surgery			
Surgical Satisfaction Questionnaire	8 questions with adjectival items (5 categories)	Adults	No
Michigan Hand Outcomes Questionnaire - Satisfaction	1 of the 6 MHQ domains, scored 0–100	Adults	No
Scoliosis Research Society-22	Satisfaction-related questions with adjectival response (5 categories)	Parents/caregivers	Yes
MODEMS - Satisfaction	9-item instrument	Adults	No
HSS ACL Satisfaction Survey	10 questions, each scored on scale of 1–5	Adults	No

There are limited satisfaction measurement tools specific to surgery and orthopedics. The Surgical Satisfaction Questionnaire is a validated tool that includes eight questions about pain control, performing daily activities, returning to work, exercising, surgical results, likelihood to make the same treatment decision again, and likelihood to recommend the surgery to someone else [45]. The Michigan Hand Outcomes Questionnaire has a subscale with questions related to patient satisfaction [46]. Similarly, the Scoliosis Research Society-22 outcome questionnaire includes satisfaction queries, and the MODEMS questionnaire includes a validated nine-item instrument to measure satisfaction [40]. Finally, HSS developed a validated and reliable ACL Satisfaction Survey (HSS ACL-SS) consisting of ten items identified by patients as being important for satisfaction after ACL reconstruction surgery [9].

Similar to expectation tools, patient satisfaction tools within the pediatric—and specifically pediatric orthopedic—literature are sparce. In general, patient-reported outcome measures are underutilized in pediatric orthopedic literature, and those that are frequently employed are often not validated or designed for a pediatric population. An exception to this is the Swedish Parent Satisfaction Questionnaire, which is a 63-question validated survey not specific to surgical care that has been used to evaluate satisfaction in pediatric orthopedic care [47•]. The Hospital Consumer Assessment of Healthcare Providers and Systems (HCAHPS) has also been used to measure satisfaction among caregivers in the outpatient pediatric orthopedic setting [48••, 49]. There is clearly an unmet need to develop and validate tools to measure patient and family satisfaction within pediatrics as a whole and pediatric orthopedics specifically.

## Relationship Between Expectations, Satisfaction, and Outcomes

Expectations have been shown to influences outcomes in the adult orthopedic literature [19] as well as in the field of medicine in general [18]. The downstream effects of psychoneuroendocrine and psychoneuroimmunologic pathways have been shown to affect biologic disease processes, which drives home the relationship between patients psychological state (expectations) and how their body responds (outcomes) [18]. There have been many studies in the adult orthopedic literature linking expectations and outcomes [3, 4, 10, 29, 50]. Patients undergoing total knee arthroplasty (TKA) with higher expectations for functional improvement had greater functional improvement on WOMAC index 6 months postoperatively [51]. Conversely, patients undergoing total hip arthroplasty (THA) who had rated improvement in pain higher as a preoperative expectation had less improvement in pain scores at 3 months [51]. Other studies in THA have found that greater preoperative expectations were associated with greater improvement on WOMAC scores at 12 months [52]. Similarly, it has been shown that greater expectations of pain improvement postoperatively in TKA and THA have resulted in higher WOMAC pain scores at 6-month follow-up [31]. Finally, THA and TKA patients who expected to have complete pain relief had better pain and function outcomes based on WOMAC and SF-36 [53]. In both rotator cuff repair (RCR) and total shoulder arthroplasty (TSA), higher expectations have also been associated with improved outcome scores with various different outcome scoring modalities [54–57]. Additionally, in the spine literature, greater expectations have also been associated with improved outcomes [58–60]. Similar findings have also been shown in patients with distal radius fractures [61].

Satisfaction in the adult orthopedic literature has also been shown to be related to patient expectations [19]. Meeting patient expectations following surgery has been linked to patient satisfaction in THA, TKA, and RCR [43, 62, 63]. In the spine literature, it has been shown that patients with greater expectations following discectomy was predictive of satisfaction [64, 65], while the effect was not seen for laminectomies [64]. The type of higher expectation in the spine literature (function versus pain) has also been linked to the degree of postoperative satisfaction [59]. As demonstrated by these results, it is imperative that the surgeon understand the patient’s expectations prior to undergoing surgery in order to either meet these expectations or have an informed discussion with the patient in order to align expectations in order to achieve high patient satisfaction.

There is a paucity of literature regarding patient expectations, outcomes, and satisfaction in the pediatric orthopedic population. The lack of literature may stem from the lack of validated measures of patient expectations in pediatric orthopedics, driving the need for further tools to evaluate these metrics. Additionally, the pediatric patient population poses a specific challenge given both the patient and caregiver expectations must be evaluated. Currently, there is one ongoing study to develop a validated measure of patient expectations prior to undergoing posterior spinal fusion for adolescent idiopathic scoliosis (unpublished data). Singleton et al. looked into satisfaction in the outpatient pediatric surgery clinic and showed that caregiver satisfaction was most closely correlated to perceived physician empathy [48••]. Sieberg et al. found that patients with greater preoperative expectations for improvements in their spinal appearance had greater satisfaction following surgery [66]. Further studies are needed to better understand the relationship between expectations, outcomes, and satisfaction.

As surgeons obtain a better understanding of the link between expectations and satisfaction, some authors have begun to evaluate whether modifying patient expectations can improve satisfaction. Padilla et al. demonstrated that discussions with patients preoperatively about length of stay can modify their expectations and increases their satisfaction following THA [67]. As more validated measures of patient expectations are developed for the pediatric population, methods for modifying these expectations can help to improve patient satisfaction.

## Conclusions

Understanding patient expectations is critical to predicting outcomes and satisfaction after elective orthopedic procedures. The majority of research has focused on adult patients, and there is limited data on pediatric patient and caregiver expectations and satisfaction. There is an established correlation between child and caregiver expectations, and satisfaction in the pediatric setting is often correlated with physician empathy. Additional research is needed to understand expectations in pediatric patients with the goal of understanding its correlation with outcomes and satisfaction after surgery.
